# Dissemination of Methicillin-Resistant Staphylococcus aureus Sequence Type 764 Isolates with Mupirocin Resistance in China

**DOI:** 10.1128/spectrum.03794-22

**Published:** 2023-01-09

**Authors:** Yinjuan Guo, Linling Xu, Bingjie Wang, Lulin Rao, Yanlei Xu, Xinyi Wang, Huilin Zhao, Jingyi Yu, Ying Zhou, Fangyou Yu

**Affiliations:** a Department of Clinical Laboratory, Shanghai Pulmonary Hospital, Tongji University, School of Medicine, Shanghai, China; b Fenyang Affiliated Hospital of Shanxi Medical University, Fenyang, China; c Department of Laboratory Medicine, The First Affiliated Hospital of Wenzhou Medical University, Wenzhou, China; University of Calgary

**Keywords:** *Staphylococcus aureus*, mupirocin resistance, molecular characteristics, ST764

## Abstract

Mupirocin, a topical antimicrobial agent, is an important component in the eradication of methicillin-resistant Staphylococcus aureus (MRSA) colonization. The molecular characteristics of 46 mupirocin-resistant MRSA (MR-MRSA) clinical isolates were analyzed by multilocus sequence typing (MLST), staphylococcal cassette chromosome *mec* element (SCC*mec*) typing, *spa* typing, and analysis of virulence genes. All 26 MRSA isolates with low-level mupirocin resistance possessed a V588F mutation in *ileS*. Among 20 MRSA isolates with high-level resistance to mupirocin, all carried *mupA*; 2 isolates also possessed the V588F mutation in *ileS*, and 1 possessed the V631F mutation in *ileS* (isoleucyl-tRNA synthetase). The majority of MR-MRSA isolates were resistant to erythromycin, clindamycin, tetracycline, ciprofloxacin, and gentamicin, but the rates of resistance to rifampin and fusidic acid were 8.7% and 6.5%, respectively. Eight sequence types (STs) were found among the 46 MR-MRSA isolates, of which ST764 was the most prevalent (76.1%). The most frequent *spa* type identified was t1084 (52.2%). The SCC*mec* type most frequently found was type II (80.4%). The most common clone among low-level MR-MRSA isolates was ST764-MRSA-SCC*mec* type II-t1084 (23 isolates), while ST764-MRSA-SCC*mec* type II-t002 (9 isolates) was the most common clone among high-level MR-MRSA isolates. Additionally, all toxin genes except the *seb* gene were not identified among ST764 isolates. Among clonal complex 5 (CC5) isolates, immune evasion cluster (IEC)-associated genes (*chp*, *sak*, and *scn*) and *seb* were present in ST764 but absent in ST5, while *sec*, *sel1*, *tsst-1*, and *hlb* genes were identified in ST5 but absent in ST764. In conclusion, the spread of CC5 clones, especially a novel ST764-MRSA-SCC*mec* type II-t1084 clone with high-level resistance to mupirocin, was responsible for the increase in mupirocin resistance. These findings indicated that the emergence of the ST764 MR-MRSA clone involves a therapeutic challenge for treating serious MRSA infections.

**IMPORTANCE** Mupirocin, a topical antibiotic that is commonly used for the nasal decolonization of MRSA and methicillin-sensitive Staphylococcus aureus in hospital settings and nursing homes, was introduced as a highly effective antibiotic against MRSA. Mupirocin acts by competitively binding isoleucyl-tRNA synthetase, thereby disrupting protein synthesis. This drug shows bacteriostatic and bactericidal activity at low and high concentrations, respectively. However, with the increase in mupirocin use, low-level and high-level resistance during nasal mupirocin treatment has been reported. In a previous study, the proportion of MRSA strains with high-level mupirocin resistance in a Canadian hospital increased from 1.6% in the first 5 years of surveillance (1995 to 1999) to 7.0% (2000 to 2004).

## INTRODUCTION

Methicillin-resistant Staphylococcus aureus (MRSA), which is a common human pathogen in nosocomial infections, has emerged as a global pathogen in hospital and community settings; it imposes a large burden on health care resources and significantly contributes to morbidity and deaths (1). A major risk factor for nosocomial infections is the extensive use of central venous catheters, heart valves, artificial lenses, and prosthetic joints (2). Moreover, colonization with MRSA has been linked to some invasive MRSA infections and greatly increases the risk of MRSA infection.

Mupirocin (pseudomonic acid A), which is a topical antibiotic that is commonly used for the nasal decolonization of MRSA and methicillin-sensitive S. aureus (MSSA) in hospital settings and nursing homes, was introduced as a highly effective antibiotic against MRSA (3). Mupirocin acts by competitively binding isoleucyl-tRNA synthetase, thereby disrupting protein synthesis (3). This drug shows bacteriostatic and bactericidal activities at low and high concentrations, respectively. However, with the increase in mupirocin use, low- and high-level resistance during nasal mupirocin treatment has been reported (3). Low-level resistance to the antibiotic (MICs of 8 to 256 μg/mL) arises by mutation of the mupirocin target, isoleucyl-tRNA synthetase (*ileS*), whereas expression of a new isoleucyl-tRNA synthesis by a plasmid *mupA* gene causes high-level mupirocin resistance (MICs of ≥512 μg/mL) (4). The *mupA* gene is typically located on mobile genetic elements and is plasmid mediated. In addition to the *mupA* gene, another mechanism of high-level mupirocin resistance, mediated by a novel locus (*mupB*), has been reported (5). The *mupB* gene (3,102 bp) shares 65.5% sequence identity with *mupA* but only 45.5% with *ileS*. In a previous study, the proportion of MRSA strains in a Canadian hospital with high-level mupirocin resistance increased from 1.6% in the first 5 years of surveillance (1995 to 1999) to 7.0% (2000 to 2004) (4, 6). There seems to be no mandated testing for MRSA colonization in China.

Two major clones, i.e., sequence type 239 (ST239)-MRSA-staphylococcal cassette chromosome *mec* element (SCC*mec*) type III and ST5-MRSA-SCC*mec* type II, were shown to be prevalent in China in the past few decades (7). The distribution of MRSA clones is geographically dynamic. In a previous study, phenotypic high-level mupirocin resistance and *mupA* were predominantly found in ST8 and ST36, while phenotypic low-level mupirocin resistance and V588F were predominantly found in ST239/ST241, as well as ST8 and ST36 (8). Rates of high-level mupirocin resistance and low-level mupirocin resistance were low (<4%) in the currently dominant UK MRSA clone ST22 and community/sporadic MRSA isolates (8).

At present, the prevalence of mupirocin-resistant MRSA (MR-MRSA) in China is limited. We determined the prevalence of mupirocin resistance among MRSA isolates from six provinces in China. We compared the MR-MRSA clinical isolates by SCC*mec* typing, *spa* typing, multilocus sequence typing (MLST), virulence genes, and antibiotic resistance profiling.

## RESULTS

### Prevalence of mupirocin resistance among MRSA clinical isolates.

Of 457 MRSA isolates tested, 46 (10.07%) were confirmed to be resistant to mupirocin (MICs of >8 μg/mL). Twenty MRSA isolates showed high-level mupirocin resistance, representing 43.48% of the MR-MRSA isolates (mupirocin MICs of ≥256 μg/mL).

Among the MR-MRSA isolates screened, 25 isolates (54.3%) were identified in Guangzhou, 13 (28.3%) in Shanghai, 4 (8.7%) in Chengdu, 2 (4.3%) in Wuhan, 1 (2.2%) in Nanchang, and 1 (2.2%) in Wenzhou. The sources of the 46 isolates included sputum (24/46 isolates [52.2%]), blood (13/46 isolates [28.3%]), and pus (9/46 isolates [19.6%]). Among the blood samples, 76.9% of the isolates were resistant to high levels of mupirocin; among the sputum and pus samples, the proportions were 29.17% and 33.33%, respectively.

The resistance of MR-MRSA isolates to 16 antibiotics is shown in Table 1. All 46 MR-MRSA clinical isolates were susceptible to vancomycin, linezolid, daptomycin, sulfamethoxazole-trimethoprim, dalbavancin, teicoplanin, and quinupristin-dalfopristin. The majority of MR-MRSA isolates were resistant to erythromycin (93.5%), clindamycin (93.5%), tetracycline (89.1%), ciprofloxacin (93.5%), and gentamicin (78.3%), but the rates of resistance to rifampin and fusidic acid were 8.7% and 6.5%, respectively.

**TABLE 1:** Antimicrobial resistance profiles of 46 MR-MRSA isolates, including high-level MR-MRSA and low-level MR-MRSA isolates and ST764 and non-ST764 MRSA isolates

Drug^*a*^	No. (%) of MR-MRSA isolates with resistance (*n* = 46)	% with resistance^*b*^					
		HLMR (*n* = 26)	LLMR (*n* = 20)	*P* for HLMR vs LLMR	ST764 (*n* = 35)	Non-ST764 (*n* = 11)	*P* for ST764 vs non- ST764
Erythromycin	43 (93.5)	100.0	89.5	0.085	100.0	72.7	0.0014
Clindamycin	43 (93.5)	96.3	84.2	0.152	100.0	72.7	0.0014
Tetracycline	41 (89.1)	92.6	84.2	0.368	94.3	72.7	0.045
Ciprofloxacin	43 (93.5)	96.3	89.5	0.356	97.1	81.8	0.073
Gentamicin	36 (78.3)	81.5	82.4	0.528	88.6	54.5	0.0025
Fusidic acid	4 (8.7)	15.8	3.7	0.152	5.7	1.8	0.200
Rifampin	3 (6.5)	7.4	5.9	0.772	0	27.3	0.000
SXT	0 (0)	0	0		0	0	
Dalbavancin	0 (0)	0	0		0	0	
Vancomycin	0 (0)	0	0		0	0	
Q/D	0 (0)	0	0		0	0	
Linezolid	0 (0)	0	0		0	0	
Daptomycin	0 (0)	0	0		0	0	
Teicoplanin	0 (0)	0	0		0	0	

a SXT, trimethoprim-sulfamethoxazole; Q/D, quinupristin-dalfopristin.

b HLMR, high-level MR-MRSA; LLMR, low-level MR-MRSA.

### Prevalence and geographical differences of mupirocin resistance determinants.

As shown in Table 2, all 26 isolates with low-level mupirocin resistance possessed a V588F mutation in *ileS*. We found that 20 isolates (100%) with high-level resistance to mupirocin (MICs of ≥256 μg/mL) were positive for *mupA*. Additionally, among 20 isolates with high-level resistance to mupirocin, 2 isolates also possessed the V588F mutation in *ileS* and 1 isolate also possessed the V631F mutation.

**TABLE 2:** Distribution of mupirocin MIC values according to resistance determinants among MR-MRSA isolates

Resistance determinant	No. of isolates	No. of isolates with mupirocin MIC of:						
		8 μg/mL	16 μg/mL	32 μg/mL	256 μg/mL	512 μg/mL	1,024 μg/mL	>1,024 μg/mL
*ileS* mutation	29	2	23	1		1	2	
V588F	28	2	23	1		1	1	
V631F	1						1	
*mupA*	20				1	2	12	5

Among the 25 MR-MRSA isolates isolated in Guangzhou, only 4 isolates carried the *mupA* gene and 22 isolates carried the V588F mutation; 21 isolates had low-level resistance to mupirocin. A total of 13 MR-MRSA isolates were detected in Shanghai, including 1 isolate (with low-level resistance to mupirocin) with the V588F mutation and the other 12 isolates (with high-level resistance to mupirocin) all with *mupA* mutations. The V631F mutation was observed only from Wuhan.

### Molecular characteristics of MR-MRSA clinical isolates.

Five different clonal complex (CC) types (CC5, CC45, CC8, CC59, and CC1) were observed among MR-MRSA isolates. CC5 (82.6% [38/46 isolates]) was the predominant type, followed by CC45 (6.5% [3/46 isolates]) and CC8 (6.5% [3/46 isolates]). Eight STs were found among 46 isolates, of which ST764 was the most prevalent, accounting for 76.1% (35/46 isolates). Eleven *spa* types (t1084, t002, t030, t1081, t992, t015, t062, t127, t264, t437, and t2460), as reported previously, were found in 45 isolates, and 1 ST764 isolate had an unknown type. The most frequent *spa* types identified were t1084 (*n* = 24), t002 (*n* = 9), and t030 (*n* = 3). The SCC*mec* type found most frequently was type II (*n* = 37), followed by type IV (*n* = 4) and type III (*n* = 3) (Table 3).

**TABLE 3:** Molecular characteristics and resistance determinants among MR-MRSA isolates

Characteristic (no. of isolates)				No. of isolates with:		MIC (μg/mL) (no. of isolates)	Sample type (no. of isolates)	Region (no. of isolates)
CC	ST	*Spa* type	SCC*mec* type	*mupA*	*ileS* mutation			
CC5 (38)	ST764 (35)	t1084 (24)	II (24)	1	24	8 (2), 16 (21), 512 (1)	Blood (2), sputum (15), pus (7)	Guangzhou (22), Nanchang (1), Wuhan (1)
		t002 (9)	II (9)	9	0	256 (1), 512 (1), 1,024 (5), >1,024 (2)	Blood (4), sputum (5)	Shanghai (9)
		t992 (1)	II (1)	1	0	1,024 (1)	Blood (1)	Shanghai (1)
		Unknown (1)	II (1)	1	0	1,024 (1)	Sputum (1)	Shanghai (1)
	ST5 (2)	t2460 (1)	II (1)	1	1	1,024 (1)	Blood (1)	Wuhan (1)
		t264 (1)	II (1)	0	1	16 (1)	Blood (1)	Shanghai (1)
	ST965 (1)	t062 (1)	IV (1)	1	0	1,024 (1)	Blood (1)	Wenzhou (1)
CC45 (3)	ST45 (2)	t1081 (2)	V (2)	2	0	1,024 (1), >1,024 (1)	Blood (2)	Guangzhou (2)
	ST508 (1)	t15 (1)	IV (1)	1	0	>1,024 (1)	Blood (1)	Guangzhou (1)
CC8 (3)	ST239 (3)	t030 (3)	III (3)	1	3	16 (1), 32 (1), 1,024 (1)	Sputum (3)	Chengdu (3)
CC59 (1)	ST59 (1)	t437 (1)	IV (1)	1	0	1,024 (1)	Pus (1)	Chengdu (1)
CC1 (1)	ST1 (1)	t127 (1)	IV (1)	1	0	>1,024 (1)	Pus (1)	Shanghai (1)

The ST764 clone spread in Guangzhou (22/35 isolates [62.9%]), Shanghai (11/35 isolates [31.4%]), Nanchang (1/35 isolates [2.9%]), and Wuhan (1/35 isolates [2.9%]), with Shanghai having the highest mupirocin resistance rate. Except for rifampin, the antimicrobial resistance rates of ST764 isolates were higher than those of non-ST764 MRSA isolates. The erythromycin, clindamycin, tetracycline, and gentamicin resistance rates of ST764 isolates were significantly (*P* < 0.05) higher than those of non-ST764 isolates (Table 1).

ST764-MRSA-SCC*mec* type II-t1084 was the most prevalent clone (24/46 isolates [52.2%]), with the *ileS* V588F mutation and low-level resistance. ST764-MRSA-SCC*mec* type II-t002 was the second most prevalent clone (9/46 isolates [19.6%]), with the *mupA* mutation and high-level resistance.

### High-level MR-MRSA and low-level MR MRSA isolates.

An aminoglycoside resistance phenotype was more frequently observed among high-level MR-MRSA than low-level MR-MRSA isolates, but no significant differences were found between them (Table 1). It is noteworthy that the rate of resistance of high-level MR-MRSA isolates to fusidic acid was 15.7%.

As shown in Table 4, among the high-level MR-MRSA isolates, 60.0% (12/20 isolates) belonged to ST764 and 10.0% (2/20 isolates) belonged to ST45, 45.0% (9/20 isolates) belonged to *spa* type t002, and 65.0% (13/20 isolates) belonged to SCC*mec* type II. Similarly, among the low-level MR-MRSA strains, 88.5% (23/26 isolates) belonged to ST764, and 92.3% (24/26 isolates) belonged to SCC*mec* type II. However, 88.5% of low-level MR-MRSA isolates (23/26 isolates) belonged to *spa* type t1084.

**TABLE 4:** Molecular characteristics of high-level MR-MRSA and low-level MR-MRSA isolates

Characteristic	Findings (no. of isolates) for^*a*^:	
	HLMR (*n* = 20)	LLMR (*n* = 26)
CC	CC5 (14), CC45 (3), CC1 (1), CC8 (1), CC59 (1)	CC5 (24), CC8 (2)
ST	ST764 (12), ST45 (2), ST1 (1), ST5 (1), ST59 (1), ST239 (1), ST508 (1), ST965 (1)	ST764 (23), ST239 (2), ST5 (1)
*Spa* type	t002 (9), t1081 (2), t015 (1), t030 (1), t062 (1), t1084 (1), t127 (1), t2460 (1), t437 (1), t992 (1)	t1084 (23), t030 (2), t264 (1)
SCC*mec* type	II (13), III (1), IV (4), V (2)	II (24), III (2)

a HLMR, high-level MR-MRSA; LLMR, low-level MR-MRSA.

### Virulence genes.

**FIG 1:**
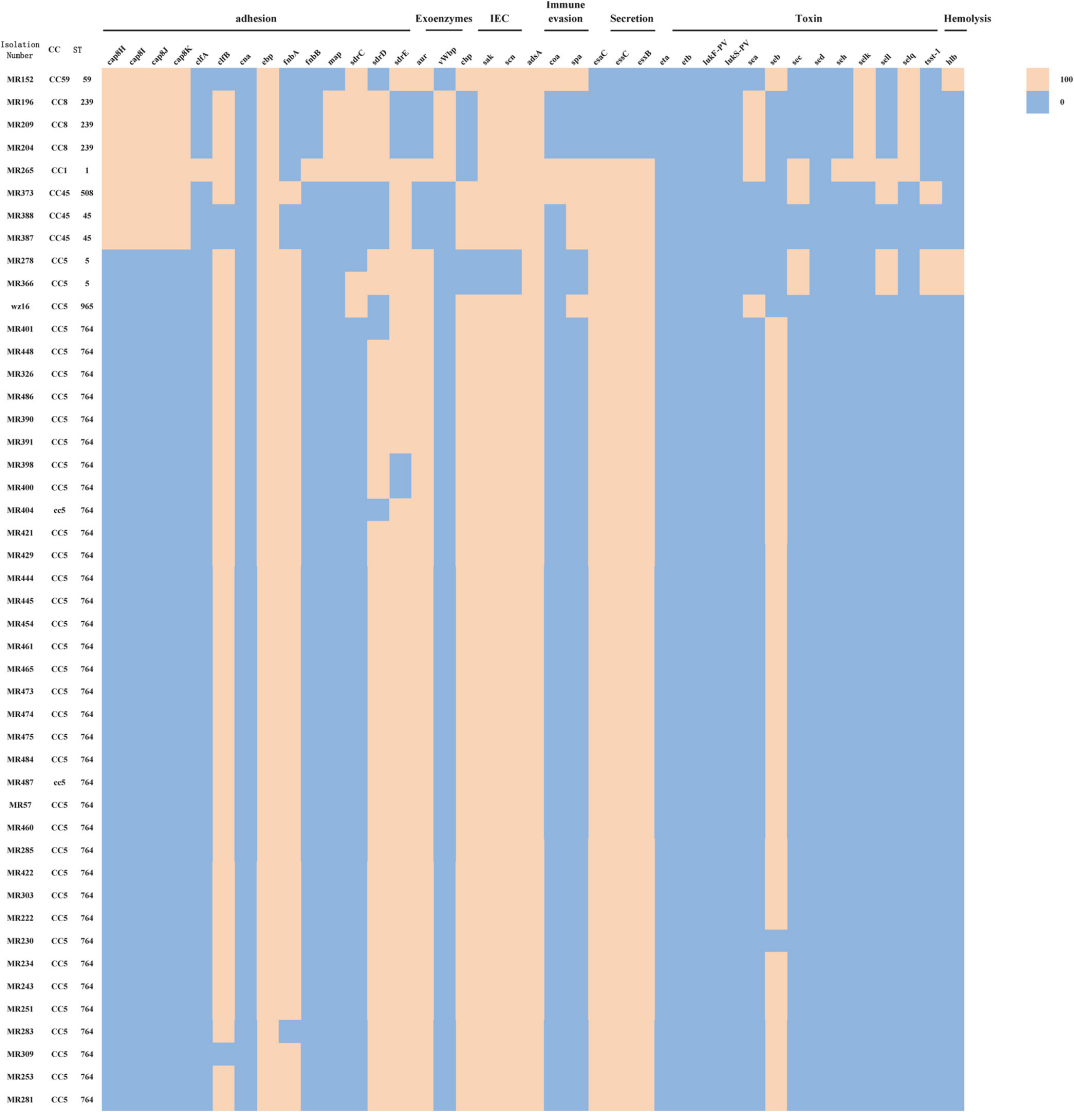
Virulence genotypes of different STs (CCs).

In this study, 81 virulence factors were analyzed for 46 MR-MRSA isolates. As shown in Fig. 1, adhesion genes, including *cap8H*, *cap8I*, *cap8J*, and *cap8K*, were not present in CC5 MR-MRSA isolates. The adhesion associated with *fnbA*, *clfB*, *aur*, and *sdrD* was more frequently carried by CC5 than by CC59 and CC8. All toxin genes except for the *seb* gene were not identified among ST764 isolates. Among CC5 isolates, immune evasion cluster (IEC)-associated genes (*chp*, *sak*, *scn*) and *seb* were present in ST764 isolates but absent in ST5 isolates, while *sec*, *sel1*, *tsst-1*, and *hlb* genes were identified in ST5 isolates but absent in ST764 isolates.

## DISCUSSION

With the increasing prevalence of MRSA strains worldwide, many states in the United States have mandated testing for MRSA colonization on admission to the intensive care unit (ICU), but there seems to be no mandatory requirement in China (9). According to literature data, MRSA colonization may even proceed faster and more easily than colonization with MSSA isolates, and the risk of symptomatic infection with MRSA is higher than that for MSSA colonization. In instances in which decolonization is to be attempted, it is important to maintain awareness of the susceptibilities of MRSA strains to antimicrobial agents that could be used for decolonization. Mupirocin is often used for this purpose. Mupirocin resistance is very important for infection control personnel who are engaged in MRSA control efforts. In this study, 10.07% of patients had MRSA isolates that were phenotypically either low-level MR-MRSA or high-level MRSA, with the prevalence of low-level MR-MRSA (5.7%) being higher than that of high-level MR-MRSA (4.4%). This is in accordance with the findings of Kim et al. (10), Chaturvedi et al. (11), and Ohadian Moghadam et al. (12), who reported that the incidence rates of mupirocin-resistant isolates were 9.5%, 18.3%, and 10.26%, respectively. Liu et al. reported that 53/803 MRSA isolates (6.6%) were confirmed to be highly resistant to mupirocin, which was significantly lower than the rate in the present study (13). However, a study in a tertiary care facility in the United States over 18 months reported mupirocin resistance among MRSA-positive patients at hospital admission in 20/591 cases (3.4%); high-level mupirocin resistance occurred in 0.62% and low-level mupirocin resistance in 2.9% (14). Also, Chen et al. stated that 26 (1.95%) of 1,333 Staphylococcus aureus clinical isolates from a Chinese hospital in Wenzhou were found to be resistant to mupirocin, including 18 (1.35%) with high-level mupirocin resistance and 8 (0.6%) with low-level mupirocin resistance (15). These differences may be attributed to the different regional policies regarding the use of antibiotics and accurate adherence to the mupirocin course for eradication.

The resistance profiles of the clinical isolates suggested that most of the MRSA isolates were resistant to most of the antibiotics. Interestingly, most high-level MR-MRSA isolates and low-level MR-MRSA isolates were resistant to antibiotics except for rifampin and fusidic acid. Vancomycin, trimethoprim-sulfamethoxazole, rifampin, dalbavancin, quinupristin-dalfopristin, linezolid, daptomycin, and teicoplanin maintained high activity against essentially all MR-MRSA isolates. No association between multidrug resistance and high-level MR-MRSA was found, as described by Perez-Roth et al. (16).

Previous studies generally showed a high level of concordance between the carriage of *mupA* and high-level mupirocin resistance (4). Point mutations in the *ileS* gene are the main mechanisms determining low-level MR. V588F and V631F are well-identified frequent mutations in IleS responsible for low-level mupirocin resistance (17). Similarly, all MRSA isolates with high-level mupirocin resistance had the *mupA* gene and all MRSA isolates with low-level mupirocin resistance possessed a V588F mutation in *ileS* in the present work. Among 26 high-level MR-MRSA isolates, 3 isolates carried both *ileS* and *mupA* genes. The result corresponded with those of Walker et al., who observed that there are populations of MR-MRSA strains involving both *mupA* and *ileS* genes (18). In addition, because genes for coresistance to macrolides, gentamicin, and tetracycline may be located alongside *mupA* on the same plasmid, mupirocin use could select for increased drug resistance in MRSA (19).

Additionally, virulence genes of ST764 were different from those of other clones. Previous studies reported that some virulence genes are found in virtually all S. aureus strains, while others are linked to specific molecular types. Compared to other STs, ST764 harbors few toxin genes except *seb*, which may help ST764 be transmitted among populations in China. S. aureus enterotoxin type B (SEB) is a superantigen. Since SEB is also considered to play a role in immune evasion upon staphylococcal infection, SEB may contribute to community infection (20). It was reported that *tsst-1*-positive CC5 isolates were associated with higher mortality rates. However, ST764 strains that belonged to CC5 did not harbor *tsst-1*.

Regarding clonal dissemination in this study, the vast majority of the molecularly analyzed MRSA clinical isolates belonged to a single clone, ST764, which was first reported in Japan (21) and was a single-locus variant of a ST5 nosocomial MRSA clone with or without the arginine catabolic mobile element (ACME) (a feature of community-acquired MRSA strains). In recent years, three studies reported ST764 S. aureus clones in China (15, 22, 23). Among them, Chen et al. found that 31.6% of mupirocin-resistant isolates were ST764 (23). Chen et at. found that ST965 was the predominant clone, accounting for 23.08% of MR-MRSA isolates, and only 2 isolates were ST764 (15). In the present study, ST764 was the most prevalent, accounting for 76.1% of isolates, and was isolated from four regions, including Shanghai, Guangzhou, Wuhan, and Nanchang, indicating clonal transmission. However, previous studies reported that, among high-level MR-MRSA isolates, 97.5% belonged to ST125 and 2.5% to ST5, being widely distributed in hospital and community settings in Spain (24). In the case of high-level MR-MRSA, clones ST22 (CC22) and ST36 (CC30) existed as the dominant UK clones between 1999 and 2009 (8). Interestingly, ST764, ST125, and ST5 are included in CC5. Dissemination of the ST764 MRSA clone, especially with multidrug resistance, should be a concern in China.

SCC*mec* type III is the predominant type in Asian countries. Similar to observations from Iran, the most prevalent SCC*mec* subtypes in China in 2010 were type III (13). Most high-level MR-MRSA isolates carried SCC*mec* type IV in Spain and the United States (25, 26). In contrast, the data in our study revealed that the most prevalent SCC*mec* subtypes were type II (79.8%).

In Spain, *spa* type t067 was the predominant type (82%), although this *spa* type was also frequently observed in the mupirocin-susceptible group (52%) (25). In China, the most frequent *spa* type identified was t030 (*n* = 23) (13). In the present study, t1084 (*n* = 24) was the predominant *spa* type, and almost all *spa* type t1084 strains (*n* = 23) were low-level MR-MRSA isolates.

The most common clone among low-level MR-MRSA isolates was ST764-MRSA-SCC*mec* type II-t1084 (23 isolates), while ST764-MRSA-SCC*mec* type II-t002 (9 isolates) was the most common clone among high-level MR-MRSA isolates. In conclusion, most of the high-level MR-MRSA isolates belonged to ST764-MRSA-SCC*mec* type II-t1084. This specific lineage is predominant in our area and is associated with resistance to aminoglycosides and macrolides. Based on our findings, we recommended future inclusion of MRSA testing in hospital laboratories. Monitoring for mupirocin resistance in MRSA, especially in ST764-MRSA-SCC*mec* type II-t1084, is necessary to monitor the usefulness of this antimicrobial drug for the treatment of MRSA infections and for infection control.

## MATERIALS AND METHODS

### Bacterial isolates.

The 46 MR-MRSA clinical isolates were isolated from six cities (Shanghai, Guangzhou, Chengdu, Nanchang, Wenzhou, and Inner Mongolia) in China between 2004 and 2020. Identification of MRSA from clinical samples was performed by using matrix-assisted laser desorption ionization–time of flight mass spectrometry (Vitek). Escherichia coli ATCC 8739 was used as a control strain for the identification. All isolates were stored at −80°C for later use. Information on all isolates is presented in Table S1 in the supplemental material.

### Antibiotic susceptibility testing.

Antibiotic susceptibility was assessed based on MIC values determined using the microdilution method. Results were interpreted in accordance with Clinical and Laboratory Standards Institute (CLSI) guidelines (27). The antimicrobial agents tested included ciprofloxacin, clindamycin, tetracycline, erythromycin, quinupristin-dalfopristin, ceftaroline, rifampin, sulfamethoxazole-trimethoprim, gentamicin, daptomycin, mupirocin, teicoplanin, linezolid, fusidic acid, vancomycin, dalbavancin, and cefoxitin. Staphylococcus aureus ATCC 25923 and Escherichia coli ATCC 29213 were used as quality control strains.

### Molecular typing methods.

All S. aureus isolates were sequenced using the NovaSeq sequencing platform (Illumina Inc., San Diego, CA), with 150-bp paired-end reads. The whole-genome sequencing (WGS) data were used for genotypic characterization and analysis of mupirocin resistance determinants. MLST was performed by submitting sequences to the MLST database (https://cge.food.dtu.dk/services/MLST). SCC*mec* typing was performed by using the SCCmecFinder database (https://cge.food.dtu.dk/services/SCCmecFinder). The *spa* typing was performed by using the spaTyper *spa* database (https://cge.food.dtu.dk/services/spatyper). Resistance-associated mutations in *ile* and *mupA* were assessed by using the ResFinder database (https://cge.food.dtu.dk/services/ResFinder). Furthermore, virulence genes were identified by using VirulenceFinder software, with a minimum query coverage of 80% and a similarity threshold value of 90%.

### Ethics statement.

This study was approved by the Ethics Committee of Shanghai Pulmonary Hospital. Because this retrospective study experimented only on bacteria and did not affect the patients adversely, the review board exempted the study from requesting informed consent.

### Data availability.

The Illumina sequences of the 46 S. aureus isolates in this study are available in the Sequence Read Archive (SRA) under BioProject accession number PRJNA881641.
